# Truncation Derivatives of the S-Layer Protein of *Sporosarcina ureae* ATCC 13881 (SslA): Towards Elucidation of the Protein Domain Responsible for Self-Assembly

**DOI:** 10.3390/molecules21091117

**Published:** 2016-08-24

**Authors:** Melinda Varga

**Affiliations:** Electronics Packaging Laboratory, Department of Electrical Engineering and Information Technology, Technische Universität Dresden, 01069 Dresden, Germany; varga@avt.et.tu-dresden.de; Tel.: +49-3514-6336-107

**Keywords:** surface layer protein, *Sporosarcina ureae* ATCC 13881, mutagenesis, self-assembly

## Abstract

The cell surface of *Sporosarcina ureae* ATCC 13881 is covered by an S-layer (SslA) consisting of identical protein subunits that assemble into lattices exhibiting square symmetry. In this work the self-assembly properties of the recombinant SslA were characterised with an emphasis on the identification of protein regions responsible for self-assembly. To this end, recombinant mature SslA (aa 31-1097) and three SslA truncation derivatives (one N-terminal, one C-terminal and one CN-terminal) were produced in a heterologous expression system, isolated, purified and their properties analysed by in vitro recrystallisation experiments on a functionalised silicon wafer. As a result, recombinant mature SslA self-assembled into crystalline monolayers with lattices resembling the one of the wild-type SslA. The study identifies the central protein domain consisting of amino acids 341-925 self-sufficient for self-assembly. Neither the first 341 amino acids nor the last 172 amino acids of the protein sequence are required to self-assemble into lattices.

## 1. Introduction

One of the most commonly observed surface structures on prokaryotic cell envelopes are crystalline arrays of proteinaceous subunits, termed surface layers (S-layers).

S-layers are composed of single protein or glycoprotein subunits which, after secretion, crystallise into two-dimensional lattices. Depending on the lattice type, one lattice unit consists of one, two, three, four or six protein monomers rendering, therefore, oblique (*p1, p2*), trimeric (*p3*), square (*p4*) or hexagonal symmetry (*p6*) to the lattice [1].

The isolated S-layer subunits maintain this remarkable intrinsic property of self-assembling into protein lattices. After detachment from the cell surface and disintegration into monomers using high concentrations of chaotropic (e.g., guanidium hydrochloride or ureae in the case of Gram-positive bacteria) [2,3] or metal chelating agents (e.g., EDTA for Gram-negative bacteria) [1,4], S-layers are able to reassemble in vitro into regular lattices identical to those observed on intact cells upon removal of the disrupting chemical. Reassembly can occur in solution, on solid surfaces, at the air-water interface or on liposomes, and the self-assembly process may lead to the formation of flat sheets, open-ended cylinders or vesicles [5]. This special property has been observed for both native and recombinant S-layer proteins.

Several studies involving Gram-positive bacteria have attempted to uncover the structure-function relationship of distinct S-layer domains. For instance, truncation analyses performed by Jarosch et al. revealed that the central S-layer part is responsible for the intrinsic self-assembly ability [6]. From their studies it become evident that the N-terminal region (aa 31-257) of the S-layer protein SbsC of *Bacillus stearothermophylus* ATCC 12980 is responsible for anchoring the protein subunits to the rigid cell wall layer, but is not required for self-assembly, nor for oblique lattice structure formation. Studies with the C-terminal truncation variants of this S-layer have revealed that more than 200 amino acids of this part (aa 880-1099) can be deleted without interfering with the self-assembly process. Similar results were obtained for the S-layer SbpA of *Bacillus sphaericus* CCM 2177 [7,8]. Whether this is a general feature of S-layers or just a particular behaviour of these two proteins, it remains to be elucidated. However, truncated S-layer structures with preserved self-assembly potential would be advantageous, because by further genetic modifications, these would facilitate the construction of various recombinant fusion proteins, therefore enhancing the properties and at the same time broadening the application potential of S-layers. Besides, the metabolic cost for producing an S-layer even in in vivo circumstances is very high; the S-layer can constitute up to 10% of the total protein of cells in the exponential growth phase [9]. Therefore, it would be advantageous to have proteins that are smaller in size and less costly for the cells to produce, allowing even their large-scale synthesis.

It was the major aim of this investigation to provide more information on the structure-function relationship of S-layer protein domains. As a study system, the S-layer protein of *Sporosarcina ureae* ATCC 13881 (SslA) was chosen. This S-layer assembles into a square lattice type with a centre-to-centre spacing of the morphological units of 12.9 nm [10]. Low stain levels and heavy metal shadowed preparations revealed that the inner and the outer surfaces of this S-layer are characteristically different. The outer surface facing the environment is smoother than the inner surface connected to the underlying cell envelope [11]. The thickness of the S-layer was found to be 6.6 nm. SslA has been reported to be an excellent biotemplate for the fabrication of highly ordered metal cluster arrays [12].

The gene encoding this S-layer (*sslA*) was sequenced, showing that the full-length SslA consists of 1097 amino acids, possesses a cleavable N-terminal secretion signal of 31 amino acids and has a calculated isoelectric point of 5.21. Adjacent to the secretion signal, two SLH domains of 43 and 45 amino acids were identified [13,14].

**Figure 1:**
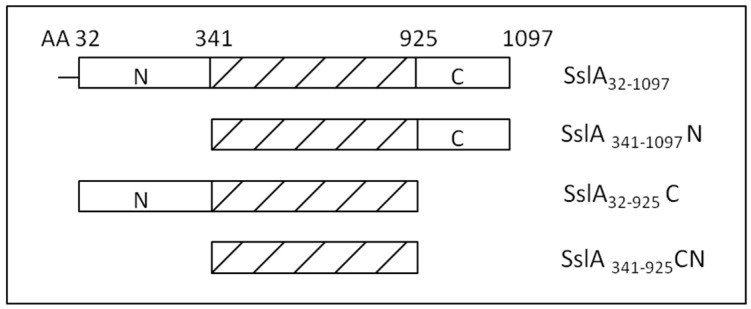
Creation of SslA truncation derivatives. By deletion of the N-terminal part of the SslA protein sequence (amino acids 1-341), SslA_341-1097_N is created. Similarly, by deletion of the C-terminal part (amino acids 925-1097), SslA_32-925_C was constructed. Finally a construct having both, the N- and C-terminals truncated (amino acids 1-341 and 925-1097) named SslA_341-925_CN was designed.

In this work, after investigating the self-assembling properties of the mature SslA protein, functional aspects of distinct segments of this S-layer are studied based on three truncation derivatives (N-terminal, C-terminal and CN-terminal truncation—Figure 1). The recombinant mature SslA and its derivatives were expressed in *E. coli*, isolated, purified and their self-assembling properties investigated by in vitro recrystallization experiments on a silicon wafer.

## 2. Results

### 2.1. Expression of the sslA Gene and SslA Truncation Derivatives, Isolation and Purification of the Various SslA Forms

The PCR products encoding the mature SslA and the SslA truncation derivatives (Table 1) were cloned and expressed in *E. coli*.

Protein expression was induced with 1 mM IPTG (final concentration) and monitored by taking culture samples before as well as 1 and 3 h following induction. The optimal temperature during expression was found to be 30 °C.

**Figure 2:**
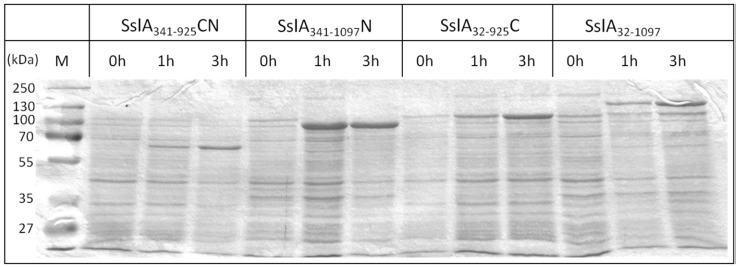
Heterologous expression of recombinant mature SslA and SslA truncation derivatives in *E. coli.* The Coomassie-stained protein gel shows expression of recombinant mature SslA_32-1097_, SslA_341-1097_N in BL21 (DE3) and SslA_341-925_CN, SslA_32-925_C in *E. coli* Rosetta Blue (DE3) whole cell lysates after 0, 1, and 3 h of induction with 1 mM IPTG (10 μg protein loaded per lane).

SDS-PAGE analysis revealed that the recombinant mature form and all three SslA truncation derivatives were successfully expressed in *E. coli* cells (Figure 2).

The gene products had the expected molecular weight, i.e., 84 kDa for the N-terminal (Figure 2, Lanes 5–7), 97 kDa for the C-terminal (Figure 2, Lanes 8–10) and 64 kDa for the CN-terminal SslA truncation derivative (Figure 2, Lanes 3–5), except the recombinant mature SslA, for which a slightly larger protein band of 130 kDa was observed in Figure 2, Lanes 11–13 (the calculated molecular mass is 114 kDa).

Mature SslA and all SslA derivatives accumulated mainly in the soluble fraction of the lysed *E. coli* BL21 (DE3) and Rosetta Blue (DE3) cells (data not shown). After isolation, they were purified by Ni-chelating affinity chromatography (all constructs contain a His tag) under native conditions. Elution fractions containing the purified proteins were dialysed against distilled water for 12 h at 4 °C.

### 2.2. Investigation of the Self-Assembly Properties of Recombinant Mature SslA and SslA Truncation Derivatives

To investigate the self-assembly properties, the purified mature SslA and SslA truncation derivatives were recrystallised in vitro on aminopropyltriethoxysilane (APTES)-modified Si wafers. To this end, 0.5 mg of the purified S-layer protein was monomerised with 5 M GuHCl for 2 hours at room temperature. Afterwards one-fifth of the monomer solution was dialysed against 10 mM Tris/HCl pH = 3 for two hours at 4 °C in the presence of the functionalised Si substrate.

**Figure 3:**
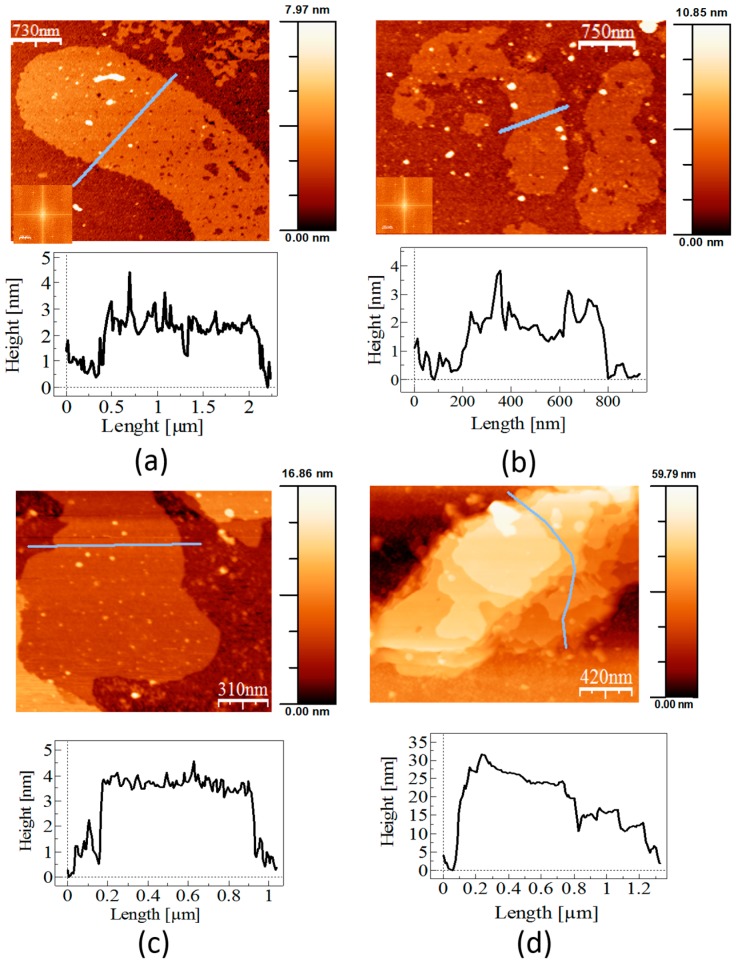
In vitro recrystallisation of recombinant SslA on a silicon wafer. AFM micrographs of the self-assembly products formed by the recombinant mature SslA and SslA truncation derivatives. (**a**) The recombinant mature SslA and (**b**) the CN-terminal truncation derivative after monomerisation with GuHCl and in vitro recrystallisation on the APTES functionalised silicon substrate at pH = 3. Both formed monolayers exhibiting square lattice symmetry; (**c**) The N-terminal and (**d**) the C-terminal truncations have self-assembled into multilayered sheets; however, the lattice structure could not be determined.

Under these experimental conditions, recombinant mature SslA (SslA_32-1097_) reassembled into crystalline, monolayered sheets (Figure 3a). The sheets are extending from 500 nm up to 6 μm in length and have a height between 2.5 and 3 nm. Fast Fourier transformation (FFT) analysis revealed *p4* lattice symmetry with a period of 12.2 nm. This value is in agreement with data obtained for the wild-type S-layer protein of *Sporosarcina ureae* ATCC 13881 [10].

Under the same in vitro recrystallizstion conditions, large, coherent and crystalline monolayers were observed for the CN-terminal SslA truncation derivative (SslA_341-925_CN) (Figure 3b). The size of this monolayer is in the order of micrometers. The height corresponds to 2.5–3 nm. The Fourier analysis clearly showed the square lattice symmetry (*p4*) with a period of 13.2 nm, which is in agreement with data obtained for wild-type SslA (*p4* and 12.9 nm) [10].

The N-terminal SslA truncation derivative (SslA_341-1097_N) self-assembled into crystalline protein multilayers, extending to sizes between 1 and 3 μm (Figure 3c). The thickness of one layer is approximately 3 nm.

Monomers of the C-terminal SslA truncation in vitro on a Si wafer formed multilayers (Figure 3d). Surprisingly, these protein layers have a measured height of 6 nm. The size of the layers is, however, smaller, being in the range of 300 nm to 1.5 μm. The lattice symmetry could not be determined for either the C-terminal or the N-terminal SslA truncation derivative.

## 3. Discussion

Up to now, several studies investigating the self-assembly properties of recombinant S-layers have been conducted. Most of these concerned bacterial S-layers that cover the cell surface of *Bacillus* strains [6,7,15]. Little is known, however, about S-layers of other species. It was the major aim of this work to extend the existing knowledge and report on the self-assembly behaviour of another S-layer protein, in particular the S-layer of *Sporosarcina ureae* ATCC 13881 (SslA). Not only the self-assembly structures this S-layer formed in vitro on a solid substrate were explored, but based on the existing genetic and structural information, functional domains were generated and analysed.

As an important prerequisite for the in vitro recrystallisation, the recombinant mature SslA (SslA_32-1097_) and all three truncation derivatives were stably expressed in *E. coli*. For several S-layers, inclusion body formation has been reported, but in this case, 50% of the proteins could be extracted in soluble form without the need of solubilisation and refolding [16]. The His-tag allowed a relatively easy purification of the SslA constructs via Ni-chelating affinity chromatography. This step is extremely important for the subsequent recrystallisation process. Pure protein solutions allow an easier identification of the self-assembly structures and do not disturb or inhibit self-assembly.

Previous attempts to recrystallise recombinant S-layers often resulted in proteins unable to form crystalline arrays [16]. However, in the case of SbsA and SbsD, recombinant S-layer proteins were produced and purified, which re-assembled into flat sheets or cylinders that exhibited the wild-type structures [17,18].

**Figure 4:**
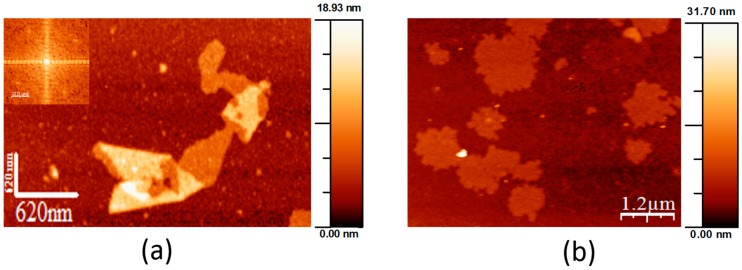
AFM micrographs of the wild-type S-layer of *S. ureae* ATCC 13881 (SslA). (**a**) Extraction of the wild-type SslA from the cell surface by a chemical treatment resulted in folded mono- and multi-layered patches that exhibit *p4* symmetry and (**b**) after monomerization with GuHCl and in vitro recrystallisation at pH = 3 on a functionalised silicon wafer, wild-type SslA self-assembled into small, rough-edged patches.

In vitro recrystallisation experiments performed in this work demonstrated that the recombinant mature SslA in vitro on APTES-functionalised silicon substrates formed crystalline monolayers in various sizes, ranging from small patches to large sheets. In comparison to the wild-type SslA isolated from the bacterial cell surface (Figure 4a), the recombinant mature SslA (SslA_32-1097_) structures are much larger. Wild-type SslA comes off from the bacterial cell surface in mono- and multi-layered (folded) patches with a length between 300 nm and 1 μm, exhibiting *p4* lattice symmetry (Figure 4a).

Applying the same in vitro recrystallisation conditions as for the recombinant mature SslA (SslA_32-1097_), wild-type SslA monomers assembled into monolayers with rough edges (Figure 4b). However, with a length between 500 nm and 1 μm, these protein layers are much smaller in size than the recombinant form. No lattice structure could be determined for these structures.

Protein recrystallisation on surfaces is a complex process. In general terms, any recrystallization process has two stages, nucleation and growth [19]. Nucleation is characterised by the appearance of small molecular clusters which, upon appropriate conditions, given that they are big enough, grow up into protein patches. By further incorporation of monomers and the aggregation of patches, layer formation occurs; however, there is always a competitive adsorption and desorption of the monomers to and from the lattice. The probability of monomers to be desorbed depends largely on the buffer pH and surface charges of the silicon surface. APTES-modified silicon substrates typically present a positively charged surface under acidic conditions. APTES reacts with silicon surfaces in such a way that the silane part gets chemisorbed on the SiO_2_ surface by reacting with the OH groups, resulting in the formation of a monolayer. The terminating NH_2_ group of APTES gets protonated by the acidic recrystallisation buffer (Tris/HCl pH = 3) to form NH_3_^+^ ions [20,21]. Besides the net positive charge, each side of the S-layer surface retains a heterogeneous distribution of positive and negative charges, which might locally affect the sticking probability of the monomers to the lattice sites or substrate (the NH_3_^+^ of APTES might interact with the COOH groups of amino acid side chains, forming covalent bonds). In this case, a small sticking probability (the ability of the monomer to adsorb rather than desorb before finding the energetic minimum and stable configuration) facilitates proper orientation and attachment of the protein monomers, giving rise to nuclei that can grow into patches and further on into an extensive layer. This explains the fact of obtaining such large S-layer sheets (~6 μm) just after two hours of in vitro recrystallisation. Plus, it has been already reported in the literature that surfaces functionalised with silane coupling agents such as APTES favour the seeding of a larger number of nucleation points than pure silicon surfaces, a fact that consequently affects the incorporation of further monomers into the protein lattices. Self-assembly is in this case independent of Ca^2+^ ions [22].

The folding of protein layers into tubular structures is very common among S-layers [3,23]. Tube formation in this case is greatly impeded due to the good binding of the SslA monolayer to the silicon surface. However, the remarkable property of isolated recombinant SslA monomers to in vitro self-assemble on silicon substrates as well as the repetitive morphological features of the obtained monolayers combined with a high porosity make them an attractive candidate for the biological functionalisation of surfaces, e.g., in the case of silicon-based sensors.

Further on, based on multiple sequence alignment data performed with sequences of S-layer proteins of *Bacillus stearothermophilus* ATCC 12980 (SbsC) and *Bacillus sphaericus* CCM 2177 (SbpA), three truncated SslA derivatives have been generated and their self-assembly properties studied in this work. As a result, truncations lacking the N-terminal 341 amino acids and the C-terminal 172 amino acids were created, plus a derivative lacking both of these SslA protein parts.

In order to define which parts of the SslA protein are subject to truncation, an alignment of the SslA protein sequence was carried out with sequences of other S-layers for which an extensive truncation analysis was already done, i.e., SbsC and SbpA. SslA showed high sequence similarity to these S-layers sequences. The sequences had the highest sequence similarity in their N-terminal parts, followed by the central protein part, while their C-terminal parts exhibited the lowest sequence similarity. This is well in line with the alignment data for most of the S-layer proteins, indicating the highest sequence similarity for the N-terminal protein part that is primarily involved in the cell wall attachment, and a moderate sequence similarity for the central part responsible for self-assembly. The C-terminal S-layer sequence is the part involved in cell wall anchoring and leads to quite diverse cell surface properties.

In vitro recrystallisation experiments have shown that all three SslA derivatives have preserved their ability to self-assemble into protein sheets. AFM experiments revealed that the self-assembly structures take the form of mono- and multi-layers with the thickness of one layer being ~3 nm. The C-terminal truncation derivative constitutes an exception, with a measured height of ~6 nm. This height difference can be perhaps related to electrostatic interactions that arise after cutting the C-terminal domain. Usually this protein domain is localised outside the lattice; therefore, it is less charged (in the case of SslA the number of hydrophobic amino acids equals the number of polar ones). By cutting it, polar amino acids will dominate the protein structure. Electrostatic interactions occurring between the polar residues might result in the folding or overlapping of certain domains, giving rise to the double height of the actual protein sheets. Another explanation would be that the structural difference is related to the hydration processes. The C-terminal part is localised outside the cell, therefore it is less charged. By cutting it, the N-terminal part would favour the formation of a thin water film, which, due to the capillary forces occurring between the AFM tip and the hydrated S-layer domain, results in a larger thickness, i.e., 6 nm. This structural difference was observed not only in the case of in vitro recrystallisation on the silicon substrate, but also when recrystallisation was done in solution (data not shown).

Drying and surface tension might cause a decrease in height for the S-layer truncation structures. It is possible that in AFM, this height is measured, resulting in layer thicknesses of ~3 nm.

Fourier analysis clearly showed the *p4* lattice symmetry for the CN-terminal SslA truncation derivative with a lattice constant of 13.2 nm (Table 1), which is in good agreement with the lattice constant reported for the authentic SslA [9].

From truncation analyses performed with SbpA, the S-layer protein of *Bacillus sphaericus* CCM 2177, it is known that cutting the N-terminal 202 amino acids and the C-terminal 237 amino acids does not affect its self-assembling properties; the S-layer was still able to form self-assembly products with *p4* lattice symmetry [7]. Indeed, truncation derivatives consisting of only the N-terminal 318, 468, 618 or 768 amino acids of the protein sequence had lost the ability to self-assemble and formed unstructured aggregates. Moreover, the deletion of the 350 C-terminal amino acids was linked to a change in the lattice type from square to oblique (*p*1).

In the case of SbsC, with the various truncated forms it was demonstrated that the protein part between amino acids 258 and 880 is necessary for self-assembly and the C-terminal 179 amino acids can be deleted without affecting the oblique lattice structure [6].

The formation of self-assembly products by the N-terminal SslA truncation derivative (SslA_341-1097_N) confirmed that the segment comprising the N-terminal 341 amino acids of SslA is not involved in the self-assembly process. This is in accordance with results obtained for SbsA and SbsC, thus confirming a common functional principle for the N-terminal parts of these S-layer proteins. The protein construct bears an N-terminal Strep-tag II and a C-terminal His_10_-tag. None of them interferes with the self-assembling ability of this derivative.

In the case of the C-terminal SslA truncation derivative (SslA_31–925_C), the self-assembling ability is also preserved. Evidently, this part of SslA is not directly responsible for self-assembly and the last 172 amino acids can be deleted without interfering with the self-assembly process and without having an impact on the lattice structure (*p*4). (A similar result was obtained for SbsC_31-880_, SbsC_31-900_ and SbsC_31-920_.)

Furthermore, deletion of both the N-terminal 341 amino acids and the C-terminal 172 amino acids still does not have a negative impact on the self-assembly property of SslA. The remaining protein part (SslA_341-925_CN) is able to form S-layer sheets, supporting the view that this central SslA protein domain is sufficient and responsible for the self-assembling properties of this S-layer protein.

In comparison to the results obtained for the other two S-layer proteins SbsC and SbpA, it can be concluded that only amino acids 341-925 of the S-layer protein of *Sporosarcina ureae* ATCC 13881 are necessary for self-assembly.

Sequence alignment results have shown that both SbsC and SbpA S-layer proteins show a high similarity to the SslA sequence [14]. This work has further proved that not only the primary structure, but also the function of the distinct S-layer domains is conserved among these bacterial species. In all cases studied, the central S-layer domain mediated self-assembly and neither the N-terminal nor the C-terminal are required for proper self-assembly and lattice formation. Hence, the SslA truncation derivatives with preserved self-assembly potential can be further engineered for the construction of recombinant, functional SslA fusion proteins that combine the self-assembly properties with a broad spectrum of specific functions (e.g., ligands, antibodies, antigens and enzymes).

In conclusion, the SslA truncation derivatives created have kept their ability to self-assemble in vitro on a Si wafer. The recrystallisation experiments have demonstrated that the central SslA protein part is sufficient for self-assembly. The results open up new possibilities for further genetic modifications of this recombinant S-layer towards interesting applications.

## 4. Materials and Methods

### 4.1. Bacterial Strains and Growth Conditions

*S. ureae* ATCC 13881 cells were grown at 30 °C in *S. ureae* growth medium I (17.01 g Na_3_PO_4_ × 12 H_2_O, 1.28 g glucose, 1.0 g yeast extract, 10 g peptone 5.28 g (NH_4_)_2_SO_4_ ) while continuously agitating at 200 rpm until OD_600_ = 1.5, which is within the logarithmic growth phase.

*E. coli* cells were grown in liquid at 37 °C under continuous agitation at 140 rpm. For selection of bacterial transformants, ampicillin was added to media (final concentration 110 μg/mL). For bacterial gene expression in *E. coli* expression strains, cultures were grown until an optical density OD_600_ = 0.4, afterwards induced with IPTG (1 mM final concentration).

### 4.2. Cloning and Expression of Gene Sequences Encoding Mature SslA (aa 31-1097) and SslA Truncation Derivatives

For PCR amplification at first genomic DNA of *S. ureae* ATCC 13881 was isolated according to the Genomic Mini kit protocol (A & A Biotechnology).

A PCR product derived from PCR amplification using the primer pair Pforp46_p4 (5′-GAC GAC GAC AAG ATG GCT GAA TTC ACA GAT GTA AAA GAC AA) and Prevp46_p4 (5′-GAG GAG AAG CCC GGT TTA AGA AGT TAC TTT TAT AAC AGG TGT ATT TAG TCA) encoding the mature SslA protein (SslA_32-1097_) was cloned into the vector pET-46 Ek/Lic (Novagen) according to the manufacturer’s instructions.

The gene encoding the N-terminal truncation derivative (SslA_341-1097_) was amplified by PCR from genomic DNA using the primer pair Pfor341Licp2 (5′-GAC GAC GAC AAG ATG ACT GGC GTT AAA AAA GCA GGA) and Prevmatp4Licp2 (5′-GAG GAG AAG CCC GGT AGA AGT TAC TTT TAT AAC AGG TGT ATT TAG TCA). The PCR product was cloned into the vector pET51b + Ek/Lic (Novagen) according to the manufacturer’s instructions.

Both constructs were transformed into *E. coli* Nova Blue Giga Single cells as a non-expression host. Plasmids from positive clones were isolated using the Nucleospin Plasmid Quick Pure kit (Macherey-Nagel) according to the manufacturer instructions.

Plasmids carrying the C-terminal truncation derivative as well as the CN-terminal derivative were constructed and supplied by P. Ryzkov [23].

For heterologous expression, the recombinant plasmids carrying the mature *sslA* gene sequence, the C-terminal and CN-terminal truncation were established in *E. coli* Rosetta Blue (DE3) strain. The N-terminal truncation derivative was transformed in *E. coli* BL21 (DE3) cells. For expression, 0.5 L of Luria-Bertani (LB) medium was inoculated with 5 mL of LB-grown preculture of the respective *E. coli* Rosetta Blue (DE3) and *E. coli* BL21 (DE3) constructs. The cells were grown at 37 °C until an optical density OD_600_ = 0.4, than recombinant gene expression was induced with 1 mM IPTG. Then, 3 h after the induction of expression, the cells were harvested by centrifugation (10,000× *g* for 10 min), washed twice with ddH_2_O and the pellet resuspended in 20 mL buffer consisting of 20 mM Tris/HCl pH = 7.9, 0.5 M NaCl, 5 mM imidazol (AppliChem), 10 mg lysozyme (New England Biolabs) and 1 mM AEBSF (AppliChem). After an incubation step of 2 h at 30 °C under continuous shaking at 300 rpm, the cell suspension was chilled on ice for 15 min. After addition of Triton X-100 (Sigma, Munich, Germany) in a final concentration of 0.5% (*v/v*), the cells were broken up by sonication (Sonopuls UW 2070). Following sonication, DNase I and Rnase A (Roth) were added to the mixture and incubated for 15 min at 30 °C under continuous shaking followed by a centrifugation step at 13,000× *g* for 10 min to separate the insoluble cell fraction. The supernatant was applied to a Ni^2+^ affinity chromatography purification column (Machery-Nagel) packed with Ni-IDA agarose His-Bind-Resin (Novagen) and charged with Ni^2+^ ions. The column was washed with two volumes of Buffer B (20 mM Tris/HCl pH = 7.9, 0.5 M NaCl and 5 mM imidazol), followed by two volumes of Buffer W1 (20 mM Tris/HCl pH = 7.9, 0.5 M NaCl and 20 mM imidazol), W2 (20 mM Tris/HCl pH = 7.9, 0.5 M NaCl and 40 mM imidazol) and W3 (20 mM Tris/HCl pH = 7.9, 0.5 M NaCl and 60 mM imidazol) each. Elution of the bound fusion proteins was done by washing the column with elution buffer (20 mM Tris/HCl pH = 7.9, 0.5 M NaCl and 1 M imidazol). Five elution fractions (5 mL each) were collected and immediately dialysed against ultrapure water at 4 °C overnight.

The SDS-PAGE of cell extracts was performed as previously described [24].

### 4.3. Investigation of the Self-Assembly Properties of the Purified Recombinant Mature and Truncated SslA Proteins

To evaluate the self-assembly properties of the SslA truncation derivatives, the proteins were in vitro recrystallised on a functionalised Si surface at a protein concentration of 0.5 mg/mL. In particular, the purified proteins were at first dissolved in 5 M GuHCl and 1/5 of the solution dialysed against Tris/HCl buffer pH = 3 for 2 h at 4 °C in the presence of an APTES functionalised silicon substrate. After dialysis, the silicon substrate was washed with distilled water, dried on air and the self-assembly structures analysed by atomic force microscopy (AFM). The AFM measurements were performed tapping mode in air using the Extended Multimode AFM with Nanoscope IIIa controller system Digital Instruments, Inc. Veeco Metrology. Silicon 130 μm long tips were used for imaging.

The images were analyzed with the help of the software WsxM (Nanotech Electronica S. L., Madrid, Spain).

### 4.4. Preparation of APTES-Silicon Substrates

Si pieces were inserted into a holder and immersed in 99% HNO_3_ for 10 min. After rinsing thoroughly with water, the Si substrates were suspended into 1 M NaOH solution again for 10 min. After this treatment, the substrates were washed in water and dried at 105 °C. In a next step, the substrates were immersed in a mixture of 19 mL ethanol, 1 mL ddH_2_O and 0.5 mL APTES solution, afterwards washed in 20 mL pure ethanol two times for 10 min and finally in distilled water for another 2 min. The functionalised substrates were dried in oven at 100 °C overnight.

## Figures and Tables

**Table 1:** Survey of the properties of the recombinant mature SslA and of the truncated SslA forms.

S-Layer Protein	Length of the Encoding Gene	Calculated M_r_ (kDa)	Mr Observed on SDS Gels (kDa)	Lattice Type	pI
SslA_32-1097_	3198	114	130	square	5.05
SslA_341-1097_N	2387	84	84	-	5.31
SslA_32-925_C	2724	97	97	-	5.46
SslA_341-925_CN	2160	64	64	square	5.42
